# Novel Bifunctional Single-Chain Variable Antibody Fragments to Enhance Virolysis by Complement: Generation and Proof-of-Concept

**DOI:** 10.1155/2014/971345

**Published:** 2014-01-12

**Authors:** Georg Huber, Zoltán Bánki, Renate Kunert, Heribert Stoiber

**Affiliations:** ^1^Division of Virology, Innsbruck Medical University, Peter-Mayr-Straße 4b, 6020 Innsbruck, Austria; ^2^Department of Biotechnology, VIBT, BOKU-University of Natural Resources and Life Sciences, Muthgasse 11, 1190 Vienna, Austria

## Abstract

When bound to the envelope of viruses, factor H (FH), a soluble regulator of complement activation, contributes to the protection against a potent immune defense mechanism, the complement-mediated lysis (CML). Thus, removing FH from the surface renders viruses, such as HIV, susceptible to CML. For a proof of concept, we developed a construct consisting of recombinant bifunctional single-chain variable fragment (scFv) based on a monoclonal antibody against Friend murine leukemia virus (F-MuLV) envelope protein gp70, which was coupled to specific binding domains (short consensus repeats 19-20; SCR1920) of FH. We used *Pichia pastoris* as expression system in common shake flasks and optimized expression in high density bench top fermentation. Specific binding of recombinant scFv was proven by flow cytometry. The recombinant scFv-SCR significantly enhanced CML of F-MuLV *in vitro* implying that FH binding to the viral surface was impaired by the scFv-SCR. This novel concept to enhance virolysis may provide a new approach for antiviral treatment.

## 1. Introduction

Monoclonal antibodies (mAbs) are undoubtedly indispensable for many regiments in cancer and antiviral therapies. In applications in which Fc-dependent effector functions are not essential, smaller fragments such as single-chain variable fragments (scFv) have several advantages over their parental antibodies. They offer rapid blood clearance and enhanced tumor penetration, which makes them a good choice for oncologic imaging [1]. Moreover, scFv are candidates for delivery of cytotoxic immunoconjugates [2]. Their efficacy had been shown as diagnostic or potential therapeutic tool for some antiviral applications [3–6]. A scFv is composed of variable fragments of the light chain (vL) and heavy chain (vH) connected by a short hydrophilic polypeptide linker providing flexibility to join the two fragments. Consequently, they are expressed as a single polypeptide and are therefore easier to produce compared to full-length antibodies. A further advantage of scFv is the lack of the Fc-portion, which is responsible for the interaction of mAbs with Fc-receptors on cells, as in its absence no Fc-mediated enhancement of infection is expected. For most applications, prokaryotic or lower eukaryotic expression systems are sufficient to generate functional scFv. Although expression of scFv in *E. coli* has already been successful [7, 8], the function of heterologous expressed scFv is often impaired by misfolding or toxicity to the host cells. Alternatively, expression by the yeast *Pichia pastoris* provides a straightforward and cost-effective microbial culturing system.

One of the first lines of defense to invasive pathogens is the complement system, a part of the innate immunity. The complement is comprised of more than 30 molecules, which act in different pathways to merge finally in the formation of the membrane attack complex (MAC). This complex perforates the pathogens surface causing disruption and thus complement-mediated lysis (CML). The activity of the complement system is tightly controlled by the regulators of complement activation (RCA). One important molecule for controlling of the complement cascade is factor H (FH). This fluid phase regulator is comprised of 20 modules, the so-called short consensus repeats (SCRs). Each SCR has a defined role within FH, one of which is the recognition of negatively charged patterns on the surface of host cells. This pattern includes sialic acids, heparan sulfates, and derivatives or glycosaminoglycans (GAGs), all of which are commonly found on mammalian cell surfaces but usually not on pathogens. FH has several domains especially for recognition of these patterns. The first contact of FH to the polyanionic surface of the host cell is mediated by the C-terminal modules, that is, SCR19 and SCR20 (SCR1920) [9]. After recognition, FH undergoes conformational changes and interacts with additional complement proteins such as factor I. This results in the inhibition of complement activation and contributes to the protection of the host cell against CML. During their budding process retroviruses, such as HIV, acquire the hostcell membrane and therefore have a similar recognition pattern for FH as cellsurface of the host [10]. Thus, binding of FH to retroviruses protects them against CML similar to the host cells [11, 12]. Interfering with this FH-binding by FH-derived SCR1920 is anticipated to displace FH from the viral surface. In the absence of FH, activated complement is insufficiently downregulated on the viral surface and therefore CML is enhanced which may result in a reduction of the viral titer [12].

In this study, we used friend murine leukemia Virus (F-MuLV), a well characterized murine retrovirus [13], and a monoclonal Ab clone 48 (mAb48) recognizing envelope glycoprotein (gp70) of F-MuLV. The parental mAb48 was used to provide antiviral specificity and to establish a scFv to which FH-derived modules were coupled. These newly generated mAb48-derived scFv fused to FH SCR-modules referred to as 48scFvSCR1920 were tested for the antiviral efficacy. Further, benchtop fermentation yeast system was optimized for upscaled production of the scFv.

## 2. Material and Methods

### 2.1. Sequence Determination of F-MuLV Env-Specific mAb Clone 48

Cells from five different batches of a mAb clone 48 producing hybridoma cell line (2.4 × 10^5^ each) [17] were harvested and total RNA was purified using RNeasy Mini Kit (Qiagen). Eluted RNA (5 *µ*L) was used as template for RT-PCR with the following profile: 10′-50°C, 5′-95°C, (15′′-95°C, 30′′-55°C–65°C for LC; 55°C–68°C for HC, 1′15′′-72°C) 35 cycles, 10′-72°C final extension (iScript One-Step RT-PCR MasterMix, BioRad) with degenerated primers designed by alignment of cDNAs of 17 different mouse IgGs with the same subtype as mAb clone 48 (IgG2a) for heavy chain and 9 for light chain, respectively, found at the NCBI's webpage. Annealing temperature increment steps were set automatically by iCycler software 3.1 (BioRad). Degenerated forward primers were constructed using the leader sequences of 5′-mRNA of light- (LC) and heavy- (HC) chain (primer mAb48-LC for: 5′-ATgATgAgTCCTgCCCAgTTCCTg-3′; primer mAb48-HC for: 5′-AGCTGGGTCTTTCTCTTCCT-3′) and reverse primers matching the noncoding 3′-region of LC and HC mRNA (primer mAb48-LC rev: 5′-TGGTGGCGTCTCAGGACCTTTGTCTC-3′; primer mAb48-HC rev: 5′-TTTGCATGGAGGACAGGGCT-3′, resp.). Transcribed and amplified cDNA was separated by agarose gel electrophoresis visualized with ethidiumbromid. Signals corresponding to the expected size were gel-extracted using DNA Gel Extraction Kit (Qiagen) and further propagated by TA-cloning into pGEM-T easy vector system (Promega). Obtained DNA was sequenced and analyzed (BioEdit) and CDR-regions were determined according to Kabat and Tai Te Wu [14].

### 2.2. Short Consensus Repeat cDNA from Mouse Liver

C57BL/6 mice were purchased from Harlan Laboratories (Ro*β*dorf, Germany) and bred and maintained free of specific pathogens in the animal facility at the Division of Virology, Innsbruck, Austria. Mice were treated in accordance with the guidelines of the “European Convention for the Protection of Vertebrate Animals used for Experimental and other Scientific Purposes” and the Austrian law. Animal experiments were approved by the ethics committee of the Austrian Federal Ministry of Science and Research (BMWF-66.011/0153-II/3b/2011). In order to collect livers for the generation of fH cDNA, isoflurane (Forane, Baxter) anesthetized animals were euthanized by cervical dislocation.

Liver cells derived from C57Bl/6 mice were strained and washed in PBS (Gibco). Aliquots of 2 × 10^5^ cells were frozen at −80°C. Total RNA was extracted (RNeasy MiniKit, Qiagen) and RT-PCR was performed using primers for regions SCR11-SCR12 (primer SCR11 for: 5′-AAAGGATCCGTAGCATCATGTGCACCAC-3′; primer SCR12 rev: 5′-TAGATCTCTCCAGTTGGTCTGTTCGAAC-3′; *Bam*HI and *Bgl*II underlined) and SCR19-SCR20 according to Kristensen's mouse factor H cDNA sequence (GenBank: AAA37759.1) [18] including suitable restriction sites and eliminating the native stop-codon at the end of SCR20 (primer SCR19 for: 5′-GGATCCTCAACAGGGAAATGTGGGCCTCCTCCACC TATTG-3′; primer SCR20 rev: 5′-TTCGAATACACAAGTGGGATAATTGATGGTGCCA TTAAT-3′). cDNA was subcloned via TA-cloning into pGEM-Teasy vector system (Promega) and confirmed by sequencing.

### 2.3. Vector Construction

SCR-modules were cloned via *Eco*RI and *Xba*I into pPICZ*α*A after modification of their flanking sequences introduced by PCR with the forward primers 5′-AAGAATTCGTAGCATCATGTGCACC-3′ and 5′-AAGAATTCTCAACAGGGAAATGTGG-3′ and reverse primers 5′-TAGATCTCTCCAGTTGGTCTGTCTAGAC-3′ and 5′-AAGAATTCTACACAAGTGGGATAATTG-3′ for SCR1112 and SCR1920, respectively (*Eco*RI and *Xba*I restriction sites are underlined).

A codon-optimized DNA sequence was synthesized (GeneArt) for 48scFv in heavy-light chain orientation flanked by restriction sites *Pst*I and *Xba*I derived from pMK-vector (standard delivery vector by GeneArt) for cloning into pPICZ*α*B resulting in pPICZ*α*B-48scFv. Additionally, all constructs for yeast expression were designed using a modular expression cassette, in which restriction sites *Bam*HI and *Bgl*II have been introduced at the 3′-end for cloning sequences for SCR11 and SCR12 (SCR1112) or SCR19 and SCR20 (SCR1920) derived from cloning vectors pGEM-Teasy-SCR1112 and pGEM-Teasy-SCR1920, respectively. Cloning of SCR-sequences into 48scFv was done in the pMK-vector (provided by GeneArt-constructs). All constructs contained coding sequences for Strep-Tag II and a His-Tag at the 3′-end. pPICZ*α*B-vectors bearing 48scFv-constructs were linearized via *Pme*I and dephosphorylated by FastAP (Fermentas) prior to transformation into X33 cells.

Both *E. coli* strain XL-2 and DH5*α* were purchased from Invitrogen and used for subcloning. Vectors for cloning and expression were lab-stock derived or purchased as indicated. Chemicals for PCR, cloning, and RNA/DNA purification were purchased from Fermentas (Germany), NEB (UK), and Qiagen (Germany). Proper cloning and plasmid integrity was confirmed by sequencing of sense- and antisense-strands and by analytical restriction digestion.

The yeast strain X33 cells was a kind gift from S. Jokiranta (Finland); pPICZ*α*-vectors were purchased from Invitrogen.

### 2.4. Expression of scFv in *Pichia pastoris*

X33 cells were plated on YPD-agarose and incubated for 48 h at 29°C. A single colony was cultured in YPD and prepared to make competent cells according to manufacturer's protocol [19]. 15 *µ*g of linearized vector-DNA was used to electroporate 30 *µ*L of competent X33 cells according to the protocol of GenePulser II (BioRad) for *Pichia pastoris*. Cells were plated on Zeocin-selective YPDS-plates for 3–8 days. Obtained single colonies were tested by PCR according to Linder et al. [20] for phenotype and the integration of scFv DNA. Test cultures were performed in 25 mL scale and analyzed by slot-blot immunoblotting. The best expressing clones were chosen for upscaling. A 50 mL overnight preculture in BMGY was grown overnight (ON) at 30°C and 160 rpm. The preculture was separated to inoculate 4 × 240 mL BMMY cultures with initial 0.1%, 0.5%, or 2% methanol. Methanol concentration was maintained by adding 0.1%, 0.5%, or 2% methanol every 24 h. Further samples were prepared with 0.8% glycerol (Gly) or 2% casamino acids (CA) during induction time. After three days postinduction cells were harvested by centrifugation.

For fermentation, a 150 mL ON-culture in BMGY was used to inoculate 1.5 L of fermentation medium. In detail, modified BSM1 (CaSO_4_ 0.23 g/L; KSO_4_ 4.55 g/L; MgSO4 × 7H_2_O 3.73 g/L; KOH 1.03 g/L; glycerol 40 g/L; 85% H_3_PO_4_ 6.7 mL) was sterilized in a Biostat Bplus MO, equipped with pH-probe, dissolved oxygen- (DO-) probe, and enriched-air gassing module controlled by MFCSwin 2.1 software (Sartorius-Stedim). After cooling down PTM1 [21] was added aseptically and temperature was set to 30°C, pH to 5, and controlled by 25% ammonium hydroxide. Airflow was kept from the beginning at 0.5 vvm and stirrer set at 500 rpm. Whole culturing was performed in a three-step mode. During batch phase, conditions were kept constant and dissolved oxygen was allowed to decrease. In the following fed-batch phase, first the stirrer was adapted to 800 rpm to keep DO over 20% and then airflow was increased to a maximum of 1.25 vvm, while glycerol feeding supplemented with 12 ml/L PTM1 was maintained at 16 ml/L/h. Glycerol fed-batch phase was kept for 4–6 h until reaching biomass concentration ranging from 120 to 200 g/L wet cell weight (WCW). During induction, airflow was supplemented with pure oxygen cascadically to a maximum of 40% enrichment. Temperature range was set to 20–30°C and pH from 5 to 3 according to experiment and the fed-batch phase (glycerol feeding) was prolonged, whereas the induction time was shortened. In general, a bolus of 0.2% methanol was given 30 min prior to methanol induction and during methanol adaption, feeding was lowered until DO reached constant levels above 20%. To control current methanol concentration substrate feeding was manually interrupted and DO-spike was monitored to occur within 30 seconds and in case of delay feeding was arrested until appearance of DO-spike. Samples were taken regularly aseptically for biomass determination and controlled for pH and scFv content by immunoblotting. Culture was harvested after 50 h–80 h of induction time by centrifugation.

For time-course analysis of either shake-flask or fermentation culture 8 *µ*L SN was mixed with 5xSDS-loading buffer, boiled, and loaded directly for SDS-PAGE and analyzed by direct staining or immunoblotting.

### 2.5. Biomass Determination

Approximately 10 mL samples from fermentation cultures were harvested aseptically and centrifuged in 15 mL Falcon tubes (BD) at 9000 g for 20 min at 4°C. After centrifugation, volume was determined via the lower meniscus and SN was stored for further analysis. Falcon tubes were placed on a towel upside-down in fixed angle to allow liquid draining for 15 min and wet cell weight (WCW) was measured.

Cell density in shake-flasks cultures was determined photometrically at 600 nm (Shimadzu) with 400-fold dilutions blanked against SN taken simultaneously from the same culture gained by centrifugation.

### 2.6. Cell Viability Assay

Cell viability of *Pichia pastoris* was assessed by propidium iodide (PI) staining. Samples (200 *µ*L of a 1 : 500 dilution) were treated with 2 *µ*L of PI stock solution (10 mg/mL, Dako), mixed, and incubated for 10 min at RT. Samples were analyzed by FACSCanto II flow-cytometer. PI-positive cells were considered as dead and PI-negative as viable. As a positive control, cells were heat damaged by 45°C for 30 min in the presence of 10% methanol.

### 2.7. Purification of Constructs

To isolate the expressed proteins from the supernatant (SN) of the yeast culture, expanded bed-adsorption (*EBA*, GE Healthcare) was performed using Streamline-chelating matrix charged with Ni or Streamline-Heparin prototype matrix was equilibrated to the manufacturer's protocol. Briefly, in case of Streamline-chelating matrix, fermentation of SN was adjusted to PBS, pH 7.4, 300 mM NaCl, and 25 mM imidazole. The fermentation SN was loaded on Streamline 25 column and washed in extended bed mode (20–22 cm) at ~200 cm h^−1^ velocity. Elution was performed in packed-bed mode with a bed height of 10,5 cm and 20–40 cm h^−1^ and buffer containing PBS, pH7.4, 300 mM NaCl, and 300 mM imidazole. In case of Streamline-Heparin SN was adjusted to 1xPBS, pH7.4 with 1/3 of NaCl content, and elution was performed with 1xPBS, pH7.4 300 mM NaCl. Fractions to 35 mL were collected and analyzed by immunoblotting.

### 2.8. Sample Treatment

Obtained samples were dialyzed in SnakeSkin tubing-membrane (Pierce). Alternatively, volume of the samples was reduced by N_2_-pressurized stirrer tank 8400 (Amicon) with 3.0 kDa MWCO membranes (Millipore) and 3.0 psi maximum pressure before dialysis.

Protein concentration of dialyzed samples was determined by bicinchoninic acid assay (BCA, Pierce) or photometric analysis (Warburg-Christian).

### 2.9. Electrophoresis and Immunoblotting

For western-blot proteins were transferred onto 0.45 *µ*m pore-sized PVDF-membranes for 30 min with 3 mA by semidry blotting. Membranes were blocked (PBS, 5% skim milk, 0.1% Tween20), washed 3 times for 5 min in PBS, 0.1% Tween 20, and stained with HRP-labeled mouse anti-poly-Histidin Ab (Sigma) or HRP-labeled mouse anti-*Strep*-Tag II (IBA) Ab in PBS, 1% BSA ON at 4°C. Membranes were then washed 3 times for 10 min in PBS, 0.5% Tween 20. For native protein content, samples were loaded directly onto PVDF-membrane by vacuum with slot-blot apparatus (Hoefer) and treated as described above. For detection, the membrane was developed with diaminobenzidine (DAB, Sigma) or by enhanced chemiluminescence (ECL) according to the manufacturer's instruction (GE Healthcare).

### 2.10. Flow Cytometric Analysis

*Mus dunni* cells (5 × 10^5^) were spread in a T75-cell culture flask (VWR) and incubated at 37°C under 5% CO_2_ in RPMI supplemented with 10% FCS and 5% L-glutamine. Cells were infected after 24 h with 100000 FFU of F-MuLV in the presence of 100 mM polybrene. Three days after infection cells were detached by 50 mM EDTA and harvested by centrifugation at 4°C. Cells were stained with 48scFv-constructs, control scFv, or parental mAb48 for 30 min on ice. Cells were washed in PBS and incubated on ice with secondary antibody FITC-labeled mouse anti-poly-Histidin Ab (Miltenyi) or goat anti-mouse-IgG (Dako). After 20 min cells were washed and kept on ice until FACS analysis. Samples treated only with labeled secondary antibodies served as staining controls. Uninfected *Mus dunni* cells were also prepared and stained as described above to prove that positive signals are due to F-MuLV infection (see Supporting Information).

### 2.11. Complement Lysis Assay

Complement lysis assay was performed as described previously [22] with the following modifications. Briefly, 10000 FFU F-MuLV in RPMI was incubated in 10% normal mouse serum (NMS), 1 mg/mL RNaseA in a total volume of 100 *µ*L 1 h at 37°C. Additionally, SCRs or 48scFv constructs were added to the sample at different molar ratios (molar extent ranging from 100x to 300x or 0.1x to 2x, resp.) corresponding to the concentration of FH. The average FH concentration is assessed with 400 *µ*g/mL [23]. Reaction was stopped on ice and samples were frozen ON at −20°C to rapture damaged virions. Next day, samples were thawed and additionally treated with 1 mg/mL RNaseA to digest released viral RNA [24]. Remaining intact virions were washed, harvested by centrifugation (1 h/16000 g/4°C) and quantified by real-time RT-PCR or by infectious center assay (see below).

### 2.12. Relative Quantification of Viral RNA by Real-Time RT-PCR

The viral RNA of the remaining particles was isolated by *Viral RNA-Easy Kit* (Qiagen) according to the manufacturer's protocol. From each sample 5 *µ*L of elution was used as template for real-time RT-PCR using the *2xRT-Mix for Probes Kit* (BioRad) in triplicate. The following profile was used: 10′-50°C, 5′-95°C, (15′′-95°C, 30′′-65°C) 35 cycles with upstream primer 5′-AAGTCTCCCCCCGCCTCTA-3′, downstream primer 5′-AGTGCCTGGTAAGCTCCCTGT-3′, and F-MuLV specific fluorogenic PCR probe 5′-[6FAM] ACTCCCACATTGATTTCCCCGTCC [TAMRA]-3′ [25].

The relative quantification of nucleic acids was performed using *Gene Expression Analysis for iCycler iQ Real-Time PCR Detection System* (BioRad).

### 2.13. Infectious Center Assay (ICA)

To determine the infectivity of the remaining viruses after the complement lysis assay, permissive *Mus dunni* cells were incubated with the SN as described elsewhere [26]. Briefly, 2.5 × 10^4^ *Mus dunni* cells were spread in RPMI on 24-well plates and incubated ON at 37°C under 5% CO_2_. The following days samples from complement lysis assay containing F-MuLV virions were added to the *M. dunni* cells at 3-fold dilutions and incubated for 3 days. Cells were then fixed in ethanol and stained with F-MuLV envelope-specific monoclonal Ab 720 [26]. To detect foci the assay was developed with HRP-labeled goat anti-mouse IgG Ab (DAKO) and 3-amino-9-ethylcarbazole.

### 2.14. Statistical Analysis of ICA

Three independent experiments with a total of six data-sets were conducted and analyzed using GraphPad Prism software. Mean values were determined and one-way ANOVA was used to compare the differences of mean distributions of multiple groups to a single control group with Dunnett's multiple comparison test to calculate significance with a *P* value < 0.05.

### 2.15. Homology Modeling and Visualization

For modeling 3D-structures of 48scFv and SCR1920, the I-TASSER server was used [15]. The model with best *z*-score value was chosen and protein interaction between 48scFv and F-MuLV envelope protein [PDB: 1AOL] [27] was calculated using ZDOCK [16]. Superimposition and visualization was performed with PyMOL [28].

## 3. Results

### 3.1. Sequence Determination of mAb48

**Figure 1:**
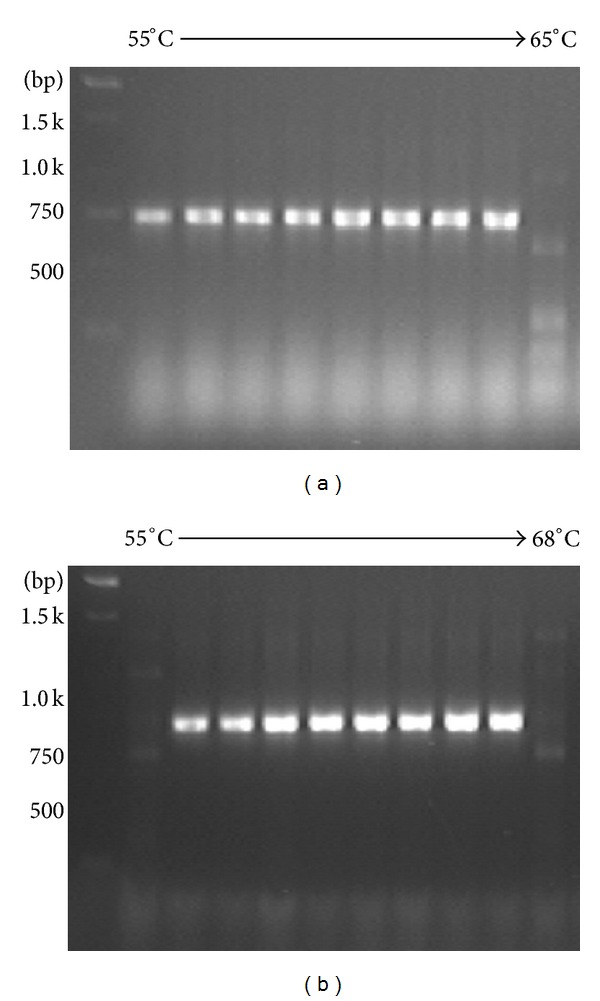
RT-PCR of total RNA isolation from mAb48-hybridoma. RT-PCR performed with increasing annealing temperature for light chain (a) and heavy chain fragment (b) followed by agarose gel electrophoresis of transcribed cDNA.

**Figure 2:**
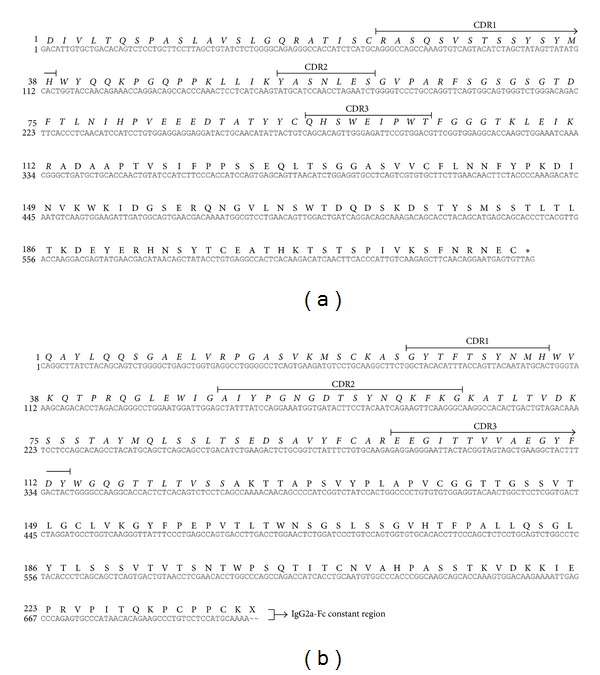
Consensus sequence of mAb48. Amino acid sequences (upper row) of heavy chain Fv (a) and light chain Fv (b) used for scFv design are in italic. CDR determined according to Kabat and Tai Te Wu [14].

First we determined the sequence of HC and LC of mAb48 [17], which targets a specific conformational epitope on F-MuLV envelope protein gp70. To generate cDNA, we used degenerated primers matching conserved mRNA-domains such as leader peptide sequence, constant CH2 region of mouse IgG2a subclasses, or LC kappa noncoding constant domains. The sequences of these mRNAs were obtained by searching in NCBI and manual alignment. Total cellular RNA was isolated from mAb48-producing hybridoma cells followed by cDNA synthesis using RT-PCR. The obtained PCR-products corresponded to the LC (Figure 1(a)) and partial HC (Figure 1(b)). The sample that exhibited a distinct signal at the highest annealing temperature was chosen for propagation assuming the highest specificity of primers to template under these conditions. LC and HC cDNA were isolated, cloned into pGEM-Teasy, and sequenced using M13-primers. Complementary determining regions (CDRs) were identified according to Kabat and Wu [14].  Figure 2 shows the complete nucleotide sequence and its corresponding amino acid sequence of LC (Figure 2(a), GenBank: JX021500) and partial HC (Figure 2(b), GenBank: JX021499), respectively. Full LC and partial HC (including CH1 and hinge-region) sequences were supposed to be sufficient to establish scFv. The beginning of constant domains after CDR3 in HC and LC was considered to be the end of an Fv (Figure 2, italic aa sequence).

### 3.2. Vector Construction

**Figure 3:**
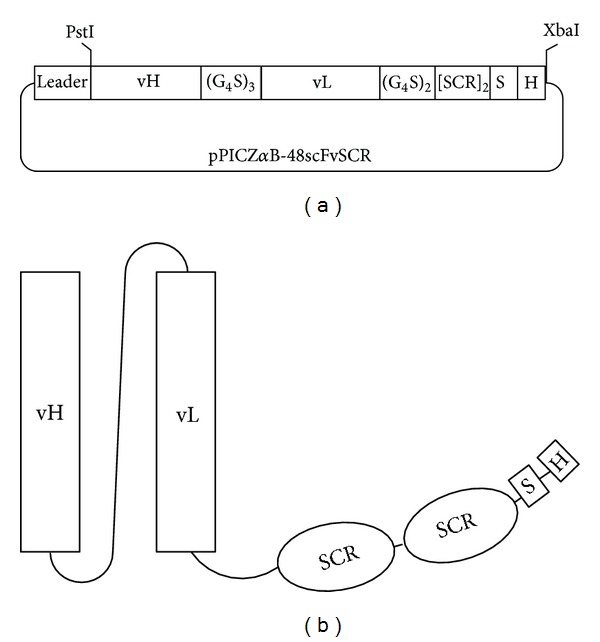
Schematic overview of 48scFVSCR1920. Expression vector pPICZ*α*B (a) for expression in *Pichia* provides a leader sequence for secretion which is cleaved prior to release of scFv. The vector contains poly-Histidine- (“H”) and Strep-Tag (“S”). Vector and final construct (b) are displayed schematically with SCR-modules.

The sequence for 48scFvSCR1920 was synthesized by GeneArt optimized for *Pichia* expression. The sequence was designed with suitable restriction sites either for exchange of the SCR-module or for direct cloning into the expression vector pPICZ*α*B via *Pst*I and *Xba*I (Figure 3(a)). The vector provides a methanol-inducible alcohol-oxidase-I (AOX1) promoter and the signal sequence of S*accharomyces cerevisiae* for secretion, which is cleaved by intrinsic proteases. This offered a native N-terminus, while a C-terminal *Strep*-Tag and *His*-Tag with adjacent stop-codon prior to the *Xba*I-cloning site was introduced to prevent additional amino acids derived from the vector. The resulting gene-product is shown schematically in Figure 3(b). After validation by sequencing, the linearized pPICZ*α*B-48scFv and pPICZ*α*B-48scFvSCR were transformed into X33 cells and positive clones were selected and propagated.

### 3.3. Expression of scFv in *Pichia pastoris* in Shake Flasks

**Figure 4:**
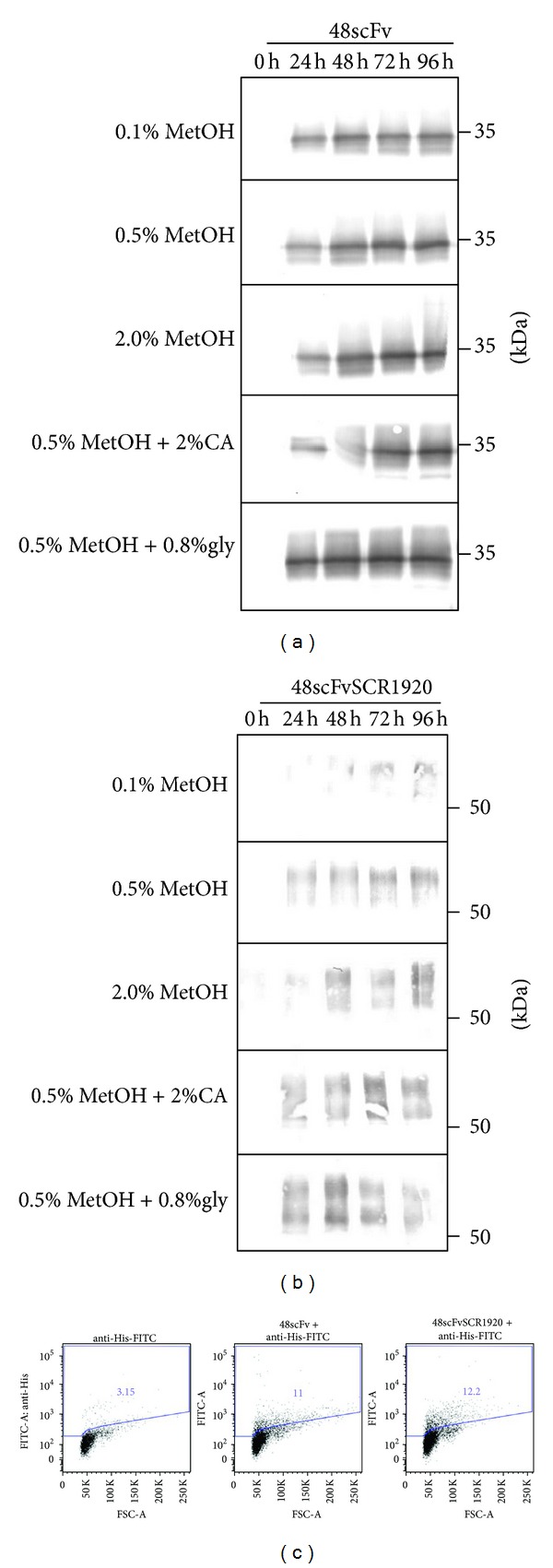
Shake-flask approach: expression analysis and binding capability. Expression of 48scFv (a) and 48scFvSCR1920 (b) in X33 cells was tested in shake flasks under different conditions and subsequently tested for binding to MuLV-env on *Mus dunni* cells using flow cytometry (c). For FACS-analysis 48scFv-constructs were derived from 0,5% methanol fed-cultures. 10 *µ*L from culture SN was loaded directly for SDS-PAGE analysis. MetOH: methanol; CA: casamino acids; Gly: glycerol. Controls for FACS-analysis, see Supporting Information S1 available online at http://dx.doi.org/10.1155/2014/971345.

Initially, we tested the expression of 48scFv and 48scFvSCR1920 in shake flask cultures under different conditions (Figure 4(a)). Our initial attempts (i.e., 0.5% methanol/24 h) revealed proper overexpression of scFv. We further investigated the influence of different methanol concentrations on the expression rate, supplementing the media with casamino acids (CA) to serve as protease-“prey” or addition of glycerol, which facilitates the carbon source adaption process [29]. Samples were periodically analyzed by western-blotting.

All culture conditions promoted the generation of 48scFv (Figure 4(a)) indicated by the increasing signal intensity over the complete induction period. The apparent molecular weight (MW) of 34 kDa corresponded to the calculated MW of 30.2 kDa. A slight difference in signal intensity was observed depending on methanol concentration, with a maximum after 72 h of induction. The effect of additional supplements, such as CA, did not improve 48scFv production. The addition of glycerol had the greatest impact on expression, with the highest yield of 48scFv.

Contrarily, the expression rate of 48scFvSCR1920 (Figure 4(b)) differed compared to that of the parental scFv. The calculated MW of 44,9 kDa for 48scFvSCR1920 appeared in all tested conditions in apparent signals slightly below 50 kDa and a smear caused by the glycosylated part SCR1920 [30]. The presence of 0.1% methanol yielded a poor expression rate at the detection limit and was observable only after 3 days of induction. The increased methanol availability resulted in higher yields, while the presence of 2% methanol resulted in a retarded expression and a signal for 48scFvSCR1920 was first detectable after 48 h of induction. All three methanol-only fed cultures showed an increase of signal corresponding to 48scFvSCR1920 over the complete induction time. The supplementation of CA had no beneficial effect on protein accumulation in the medium. The additional carbon source glycerol led to an expression maximum after 48 h of induction.

The binding capability of 48scFv and 48scFvSCR1920 to gp70 of MuLV was assessed by flow cytometry analysis with F-MuLV infected *Mus dunni* cells expressing gp70 on the cell surface. As staining controls served non-infected cells; the infected cell population was identified using the monoclonal antibody clone 48 (See Supporting Information S1). As shown by FACS analysis, both the parental 48scFv and its modified version 48scFvSCR1920 had a comparable binding capability (Figure 4(c)) and bound specifically to cells infected with F-MuLV (Supporting Information S1).

### 3.4. Expression of scFv in *Pichia pastoris* in Fermenter Cultures

Although 48scFvSCR1920 could be produced using shake flasks, the yield was too poor to perform several lysis assays, including serial dilutions of 48scFvSCR1920, from a single batch. Therefore, we tested expression of 48scFvSCR1920 within fermenter cultures, since fermentation of *Pichia pastoris* may achieve higher expression levels relative to shake flasks [31]. *Pichia pastoris* is able to grow in a range between pH 3 and 7 [32]. To avoid potential damage of recombinant scFv by proteases, we compared the induction at pH 5 and pH 3, since it has been reported that released proteases are less active at certain pH values [33]. We also analyzed the effect of lowered temperature on protein production. Generally, most recombinant protein production in *Pichia pastoris* is performed at 30°C revealing optimal for growth or biomass accumulation. Detrimental effects on protein expression above 32°C are known [21], but no extensive investigation has been performed using *Pichia pastoris* for recombinant scFv production at low temperatures. Li et al. showed that lower temperature increased cell viability and decreased protease activity [34]. To obtain comparable experiments precultures were adjusted to equal cell densities prior to inoculation of the fermentation broth. The analyses of the experiments were performed similar as those described for the shake flask experiments.

**Figure 5:**
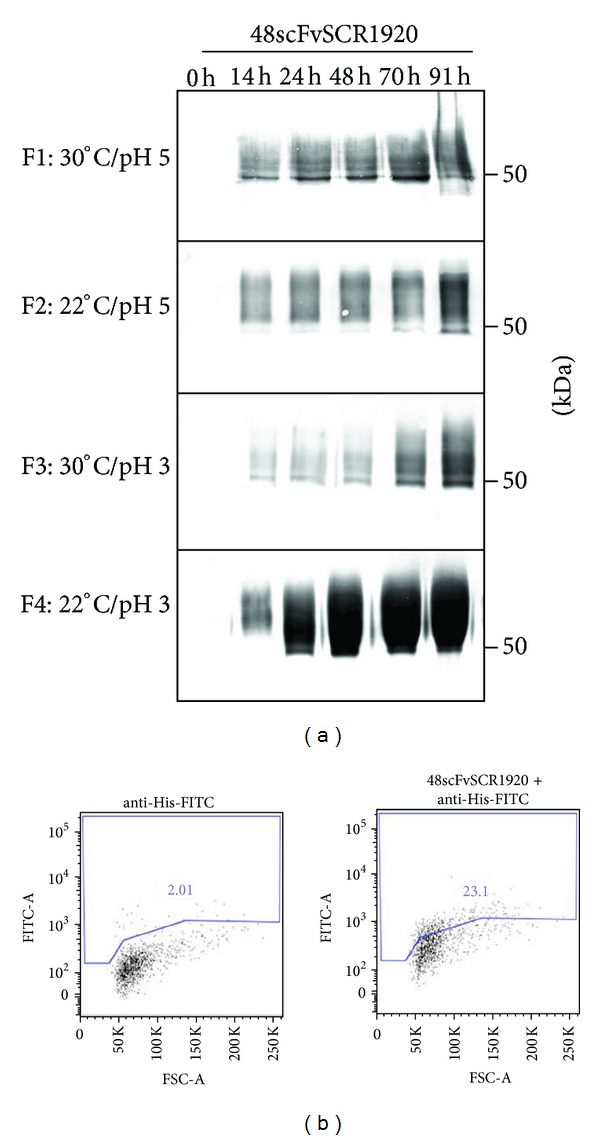
Benchtop fermentation: expression analysis and binding capability. Expression of 48scFvSCR1920 in X33 cells was tested using high-density fermentation (a) under different conditions and subsequently tested for binding to MuLV-env on *Mus dunni* cells using flow cytometry (b). For FACS-analysis 48scFvSCR1920 derived from F4 was used. 10 *µ*l from culture SN was loaded directly for SDS-PAGE analysis. F: fermentation run. Controls for FACS-analysis, see Supporting Information S1.

At all tested conditions, 48scFvSCR1920 was expressed after 14 h of induction and the signal intensity for 48scFvSCR1920 increased over the complete period of induction (Figure 5(a)). The fermentation at 30°C and pH5 (F1) showed a slight increase in signal intensity compared to the fermentation performed with the same pH and reduced temperature (F2) or the same temperature and pH 3 (F3). A boost in expression intensity was obvious at lower temperature and an induction pH of 3 (F4). After 48 h induction time, a maximum protein production was reached and maintained. The yield under the chosen conditions of fermentation F4 was 7-fold compared to F1 producing the least amount (Table 1) with no correlation between temperature and pH or biomass accumulation.

The binding capability of 48scFvSCR1920 to the envelope protein of MuLV as shown in Figure 5(b) was confirmed by flow cytometry analysis under similar conditions as described above. For controls see Supporting Information S1.

### 3.5. Complement-Mediated Lysis of F-MuLV and the Infectious Center Assay (ICA)

**Figure 6:**
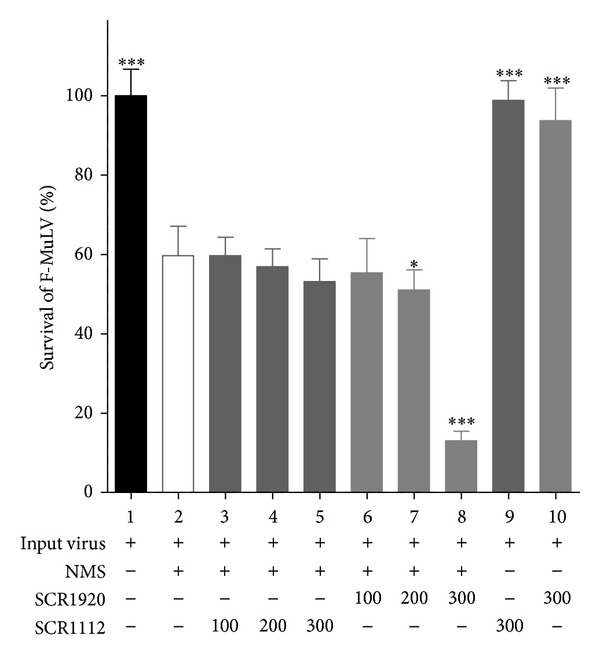
Relative quantification of viral RNA. F-MuLV was lysed in presence of NMS as a source of complement and SCR-modules in a 100–300-fold molar ratio compared to FH (100, 200, and 300 corresponding to 37 *µ*g, 75 *µ*g, and 112 *µ*g, resp.). Remaining viral RNA was quantified by real-time RT-PCR. Virus-specific RNA was detected by FAM-probes. Relative quantification was performed by the iCylcer's software.

**Figure 7:**
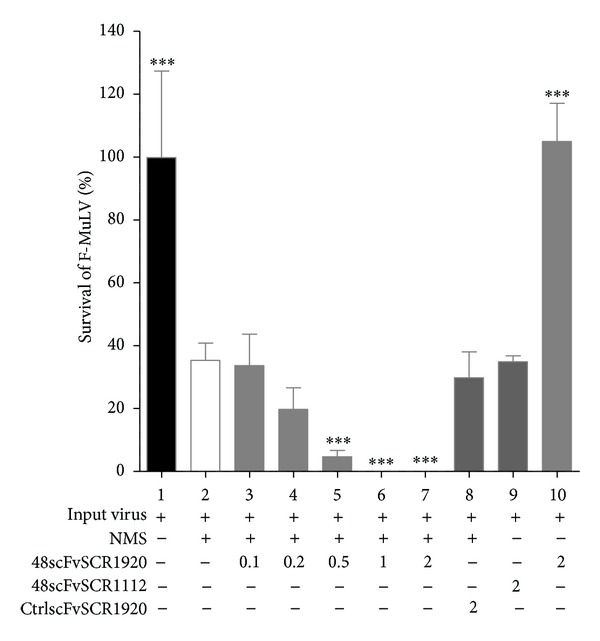
Infectious center assay (ICA). F-MuLV was lysed in presence of NMS as a source of complement and 48scFvSCR1920 in 0.1-, 0.2-, 0.5-, 1- and 2-fold molar ratio compared to FH (corresponding to 0.16 *µ*g, 0.32 *µ*g, 0.8 *µ*g, 1.6 *µ*g and 3.2 *µ*g, resp.). After lysis the amount of remaining infectious viral particles was determined by an ICA. Controls for the single-chain moiety (CtrlscFv:anti-*β*Gal-scFvSCR1920) and for the SCR-moiety (48scFvSCR1112) were added in 2-fold molar ratio to FH (3.2 *µ*g both). Summary of 6 data-sets was analyzed by using 1-way ANOVA and compared with “Dunnett's Multiple Comparison Test” performed by GraphPad Prism 5-software.

For virus lysis assay we incubated F-MuLV with normal mouse serum (NMS) as source of complement and FH in the presence of increasing concentrations of SCR1920. The central modules of FH (SCR1112) were used as control, since this region of FH has no known binding or enzymatic activity [9]. Addition of NMS as a source of active complement induced about 40% of virolysis (Figure 6, lane 2). As expected, CML was not further enhanced by the control SCR1112 at all concentrations tested (Figure 6, lanes 3–5). In contrast, the presence of the C-terminal modules SCR1920 increased the virolysis significantly in a dose-dependent manner (Figure 6, lanes 6–8). Samples without NMS and the highest concentration of FH-derived SCRs were used as controls to exclude any direct cytotoxic effect of the SCRs on the virus (Figure 6, lane 9 and 10). Next, we analyzed the effect of 48scFvSCR1920 in virolysis assays. CML was performed as described above. The remaining amount of infectious viral particles after treatment with NMS was determined by infecting permissive *Mus dunni* cells as described in the Material and Method section. Again, the addition of NMS reduced the infections titre substantially (Figure 7, lane 2) and only 48scFvSCR1920 was able to significantly reduce the amount of infectious virions significantly at a molar ratio of 0.5 compared to the amount of FH present in the NMS (Figure 7, lanes 3–7). As control for the SCR-moiety, 48scFvSCR1112 was used which had no effect on the CML assay. Also the unrelated scFv against*β*Gal (kindly provided by A. Ejaz) linked to SCR1920 was ineffective (Figure 7, lane 8). Again, a sample without NMS was used to exclude any complement-independent cytotoxicity of 48scFvSCR1920 on F-MuLV (Figure 7, lane 10).

### 3.6. Homology Modeling and Visualization

**Figure 8:**
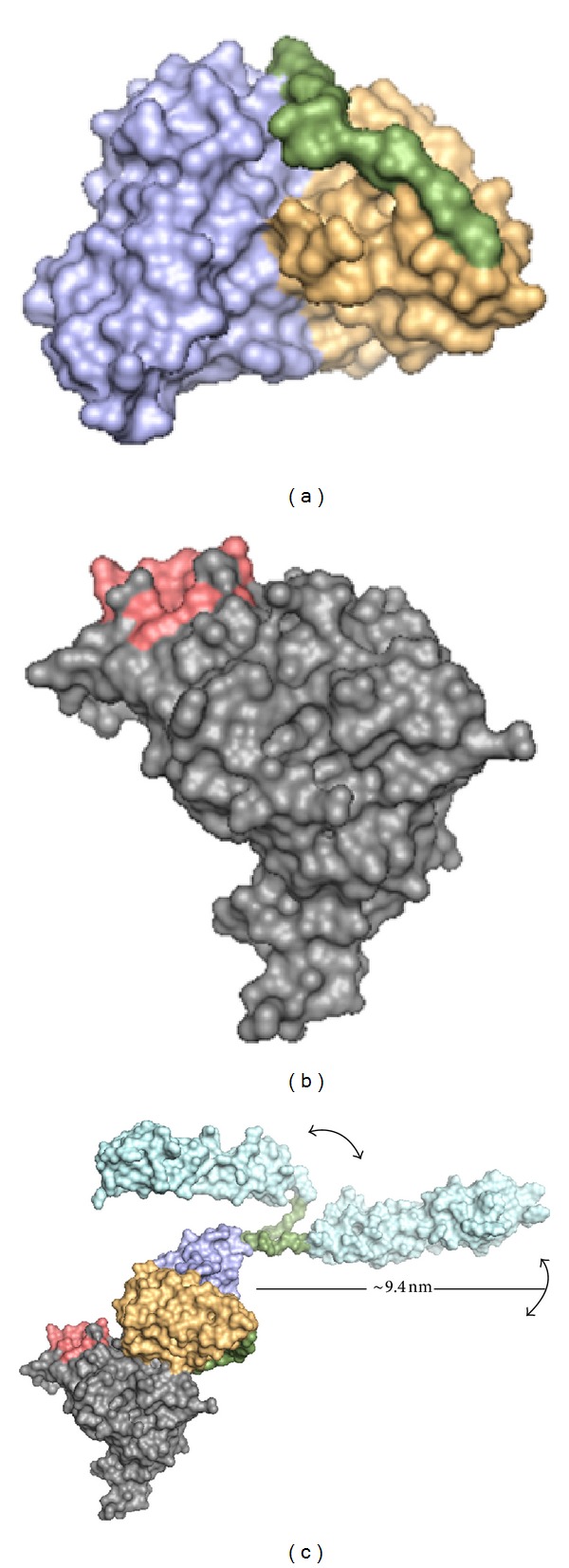
*In silico* analysis of binding 48scFv to envelope-protein of F-MuLV. (a) LC-Fv (purple) and HC-Fv (yellow) of 48scFv are connected by a (G_4_S)_3_-linker (green). (b) F-MuLV-gp70 (grey, data from pdb: 1AOL) and the main binding pocket (red) to its cellular receptor. (c) Docking calculation of 48scFv and gp70. Two SCR1920-models (light blue) were superimposed to the C-terminus of 48scFv illustrating the flexibility of the GS-linker (green) and the probable covering area (arrows). 3D-modelling of 48scFv (a) and SCR1920 ((c), light blue) was performed using I-TASSER [15]. Docking calculation of 48scFv and F-MuLV-gp70 was performed by ZDOCK [16].

To visualize how the recombinant 48scFvSCR1920 may bind to gp70 of F-MuLV, we performed homology modeling of 48scFv using I-TASSER (Figure 8(a)) and putative complex formation by docking calculation (ZDOCK) with F-MuLV envelope protein gp70 (Figure 8(b)). Fass et al. [27] analyzed F-MuLV envelope protein by crystallography [PDB: 1AOL]. They identified a putative hydrophobic binding pocket of gp70 (Figure 8(b), red) to its cellular receptor mCAT1 [35]. The overall binding interface of gp70 and mCAT1 is relatively large (1300 Å^2^) with 30 amino acids including an essential residue W102, making up the binding pocket as identified by Fass and coworkers [27]. The docking calculation of gp70 and 48scFv revealed binding of 48scFv distant from putative binding pocket. For illustration the model of the calculated SCR1920 was superimposed to the C-terminus of 48scFv (Figure 8(c), light blue). The illustration shows two different positions for the SCR1920-model indicating the linker's flexibility (Figure 8(c), green).

## 4. Discussion

Here we show that *Pichia pastoris* is a suitable system for the expression of scFvs linked to FH-derived SCRs. Both the scFv and the SCR remain functionally active in this expression system. In contrast, *E. coli *did not promote the production of functional 48scFvSCR1920 (data not shown) but allowed for initial analysis of the scFv construct. The length of the linker between HC- and LC-variable fragments and the orientation of the variable fragments, both of which may influence expression, stability and functionality, that is, binding capacity [36, 37], were examined with 48scFv produced in *E. coli.* Evaluation by FACS-analysis identified the optimal construct with a 15aa linker connecting the variable fragments in variable heavy-variable light chain orientation (data not shown). As *E. coli* did not produce functional 48scFvSCR1920, the heterologous expression in *Pichia pastoris* was tested.

The methylotrophic yeast *Pichia pastoris* provides several advantages for heterologous protein expression including their posttranslational protein processing, notably glycosylation and disulphide-bond formation. Single-chain antibodies have already been successfully produced in *Pichia pastoris *[29, 38].

To generate 48scFv-constructs, the initial experiments using shake flasks were not appropriate (Figures 4(a) and 4(b)). Since the signal intensity for 48scFv increased over the complete induction time we assumed that no proteases were released which potentially could degrade 48scFv-constructs. For that reason, additional CA as protease-prey provided no advantage. The amount of available carbon corresponded with the accumulated biomass as well as the increased signal intensity (Supporting Information S2). This is consistent with the increased signal intensity after 24 h of 48scFv in mixed-fed culture (Figure 4(a)). It is reported that mixed feeding of glycerol and methanol in *Pichia* cultures can improve yield [39]. We suggest that the additional glycerol boosted biomass accumulation and therefore detectable amounts of scFv appeared earlier compared to methanol-feeding only. Remarkably, 2% methanol feeding was not cytotoxic for the cells expressing 48scFv (see Supporting Information S2), although in shake flasks methanol uptake or any other conditions are difficult to control. For 48scFvSCR1920 a similar expression profile was observed and increased availability of carbon and accumulated biomass correlated with 48scFvSCR1920-production. In cultures fed with 0.5% methanol, signal intensity increased over time, while the 2% methanol fed culture the signal intensity reached a maximum around 48 h after induction. This correlates with the increased mortality rate of about 9% (see Supporting Information S3), suggesting that 2% methanol feeding resulted in toxic concentration after 48 h of induction. A possible retarded methanol-adaption may result in local toxic concentration by repeated feeding every 24 h. This concentration may cause cell death. Subsequently the release of proteases from dying cells may cause no further increase in 48scFvSCR1920 production. A similar signal fluctuation appeared in the mixed-fed cultures (Figure 4(b)). The additional glycerol resulted in increased biomass (Supporting Information S2), which may have also affected culturing conditions negatively in respect to aeration, starving after carbon consumption or heat development and led to potential cell death. We assume that scFv-production by increased biomass cannot counteract the negative impact of proteases which may account for the decreased signal intensity after 48 h (Figure 4(b)). The functionality of 48scFv is not impaired by the additional SCR-modules reflected by similar binding capacity to F-MuLV infected cells for both 48scFv and 48scFvSCRs (Figure 4(c)). While the yeast *Pichia pastoris* culture system successfully produced functional recombinant scFv, the yield of 48scFvSCR1920 produced in shake flasks did not fulfill our requirements. Thus, we introduced benchtop fermentation. To optimize 48scFvSCR1920 production by fermentation we tested conditions, which cannot be controlled during shake-flask culturing, that is, pH, temperature or feeding conditions. *Pichia pastoris* promotes high cell-density fermentation and offers several advantages to increase the level of heterologous expression. One important fact is the improved transcription rate of *AOX1 *promoter under growth limiting conditions compared to culturing yeast in shake-flasks [40]. The ability of *Pichia pastoris* to express scFv in shake flasks at pH 6 cannot be extrapolated to upscale into fermentation under similar conditions. Initial attempts with fermentation run at pH 6, 6.5, and 7 revealed no protein expression in our hands (data not shown), although positive scFv-production has been reported under these conditions [41, 42]. Our results suggest that culturing at 30°C has a negative effect on viability at high density fermentation (Figure 5(a), F1 and F3). Most probably the release of proteases from dying cells may have diminished the overall yield of secreted proteins. We identified the optimal conditions for 48scFvSCR1920-production at pH 3 and 22°C (Figure 5(a), F4) assuming fewer proteases due to temperature and optimal pH for its production with confirmed functionality indicated by FACS-analysis (Figure 5(b)).

FH plays an important role in recognition of self-surfaces and therefore shields hostcells from self-destruction by CML [23]. During the budding process, retroviruses acquire the host-derived membrane including proteins which bind fH [12]. Thus, fH accumulates on the viral surface protecting the pathogen from complement destruction. The C-terminal modules SCR19-20 of FH are thought to initiate the initial contact with the cell or viral surface, respectively [9]. Thus, 48scFvSCR1920 is thought to occupy binding domains of FH specifically on the viral surface targeted by the antibody fragment. Thereby MAC-formation can take place leading to lysis of viral particles or infected cells. In a first experiment, we tested the ability of the SCR-modules 19-20 (SCR1920) to interact with FH on the viral surface assessed by a viral lysis assay followed by a relative quantification of remaining viral RNA by real-time RT-PCR. Figure 6 shows that SCR1920 is able to enhance CML upon 200-fold excess compared to FH in the sample. FH itself has a low affinity to GAGs which is compensated by its high concentration in serum [9]. Due to the law of mass action an excess of SCR should be able to displace FH from the viral surface, since FH and SCR1920 have almost comparable affinity to negative charged surfaces [30]. Therefore, such a high concentration of SCR1920 is needed. In order to reduce the concentration of SCR-modules necessary to interfere with FH binding, the affinity of SCR1920 was artificially increased by connecting the modules to a virus-specific antibody fragment, which targets the SCR to the surface of the virus. In a tumor setting using cells of patients with chronic lymphocytic leukemia, lysis was specific for the tumor cells in the presence of a tumor specific antibody and SCR18-20 [43, 44]. Even at such high concentrations of SCR, neither T cells, nor erythrocytes, BHK-21 fibroblasts nor Colo-699 epithelial cells were affected [43, 44]. Therefore we assume that in humans not-infected cells were also not lysed by this approach. To further reduce putative side effects and enhance efficacy, we generated the 48scFvSCR1920 construct to displace FH from the viral surface. We assessed the CML-enhancing potential by a viral lysis assay followed by an ICA to determine the titer of the remaining infective viral particles. Our data reveal that a specific concentration corresponding to a 48scFvSCR1920/FH ratio of 0.5 was sufficient to induce virolysis. Thus, 600-times less 48scFvSCR1920 was necessary to induce efficient CML to reduce the amount of remaining infectious viral particles when compared to uncoupled SCR1920 (Figure 6, lane 8, versus Figure 7, lane 5). The control groups, 48scFvSCR1112 and CtlrscFvSCR1920, exhibited no beneficial effect on virolysis (Figure 7, lanes 8-9) in a 2-fold ratio to FH (indicated by “2” in Figure 7).

The modeling of 48scFv revealed a proper epitope-binding site (Figure 8(a)) consisting of the CDRs of variable LC and HC. The complex-calculation of 48scFv and gp70 shows docking of 48scFv to F-MuLV (Figure 8(c)). The main binding pocket of F-MuLV to its cellular receptor remains unaffected (Figure 8(b), red) and is consistent to the data of the ICA in presence of 48scFv-SCR-constructs without NMS, since no reduction in infectivity is revealed.

These data suggest that 48scFv-SCR constructs may not interfere with the binding area on either the monomeric or trimeric form of gp70 and do not hamper infection at all. The superimposed SCR1920-models on 48scFv's C-terminus (Figure 8(c), light blue) picture the potential covering area on the viral surface and thus, the probable interfering with FH-binding. Förster et al. [45] investigated the 3D-structure from ecotropic MuLV and Env. Their data allow calculating the putative covering area of 48scFvSCR1920: the diameter of MuLV-virions is given with 100 nm, which corresponds roughly to 31.500 nm^2^; 50 Env-trimers in average are present on the viral surface. Three 48scFvSCR1920 bound on each Env-trimer (trimer-radius 5 nm plus 9.4 nm SCR-radius) result in a covering area of 650 nm^2^/trimer corresponding to 32.500 nm^2^/virion. These data indicate the theoretical possibility to cover a virion completely.

The SCRs at the C-terminal end of the 48scFv are also distant from either the binding pocket of F-MuLV or from the binding interface of the 48scFv itself to gp70. It is reported that the parent antibody clone 48 slightly affects infection of about half a log [46] suggesting that mAb48 binds to a relevant region on gp70 for binding its cellular receptor. Detailed information about mAb48 and its target (i.e., epitope mapping) for further analysis has not been done yet. The 48scFv binds distant from the main binding pocket and may also show reduced affinity compared to its parental antibody [47], since scFv usually exhibit reduced avidity due to their monovalent structure. Nevertheless, this *in silico* binding data stresses the potential of the SCR-modules and excludes neutralizing effects of the 48scFv-moiety of the scFv-SCR constructs. However for conclusive data, X-ray from crystallography and NMR-data are compulsory.

In this study we generated a novel recombinant bifunctional scFv by the methylotrophic yeast *Pichia pastoris*. The yield of the obtained product (48scFvSCR1920) by conventional shake flask cultures did not meet the requirements for further experiments. However, the production of 48scFvSCR1920 by the yeast system was improved by fermentation. We also could show that *Pichia pastoris* is an easy and suitable tool for our bifunctional recombinant scFv expression. Functional 48scFv recognized its viral target independent of the modification with SCR-modules. Reliable upscaling by *Pichia*-high-density fermentation has provided sufficient amount for expanded experiments. We further showed for the first time the powerful antiviral effect of a 48scFvSCR1920 construct most likely mediated by the displacement of FH specifically from the viral surface. This newly established principle might be a basis for further investigations to use FH-derived SCRs linked to specific Abs not only for antiviral but also for cancer therapy [43, 44].

## Supplementary Material

Supporting information S1 shows the binding of 48scFv and 48scFvSCR1920 including all controls analyzed by FACS. Supporting information S2 illustrates growth curves from shake-flask cultures of Pichia expressing 48scFv or 48scFvSCR1920 under different feeding conditions over time. Supporting information S3 summarizes cell viabilities of Pichia expressing 48scFv or 48scFvSCR1920 after 4d of induction under different conditions.

## Figures and Tables

**Table 1:** Summary of fermentation runs producing 48scFvSCR1920.

Fermentation (F) number	X33 expressing	pH during induction	Temperature during induction	WCW^1^ at induction	WCW end of run	yield^2^ [mg/L]
F1	48scFvSCR1920	5	30°C	250	294	18
F2	48scFvSCR1920	5	22°C	177	238	65
F3	48scFvSCR1920	3	30°C	182	440	56
F4	48scFvSCR1920	3	22°C	208	297	130

^1^WCW: wet cell weight; ^2^yield after first purification round using expanded bed adsorption/Streamline-chelating matrix, yield concentration corresponds to mg per L culture volume.

## Abbreviations

scFv: Single chain variable fragment
FH: Factor H
CML: Complement-mediated lysis
SCR: Short consensus repeat
F-MuLV: Friend murine leukemia virus
NMS: Normal mouse serum.
