# Supplementary material for: Novel Bifunctional Single-Chain Variable Antibody Fragments to Enhance Virolysis by Complement: Generation and Proof-of-Concept
Source: Biomed Res Int. 2014 Jan 12;2014:971345. doi: 10.1155/2014/971345 (PMC3913500; doi:10.1155/2014/971345)
Supplement: Supplementary file 1 — Supporting information S1 shows the binding of 48scFv and 48scFvSCR1920 including all controls analyzed by FACS. Supporting information S2 illustrates growth curves from shake-flask cultures of Pichia expressing 48scFv or 48scFvSCR1920 under different feeding conditions over time. Supporting information S3 summarizes cell viabilities of Pichia expressing 48scFv or 48scFvSCR1920 after 4d of induction under different conditions. [file 971345.f1.pdf]

supplement data to figure 4:

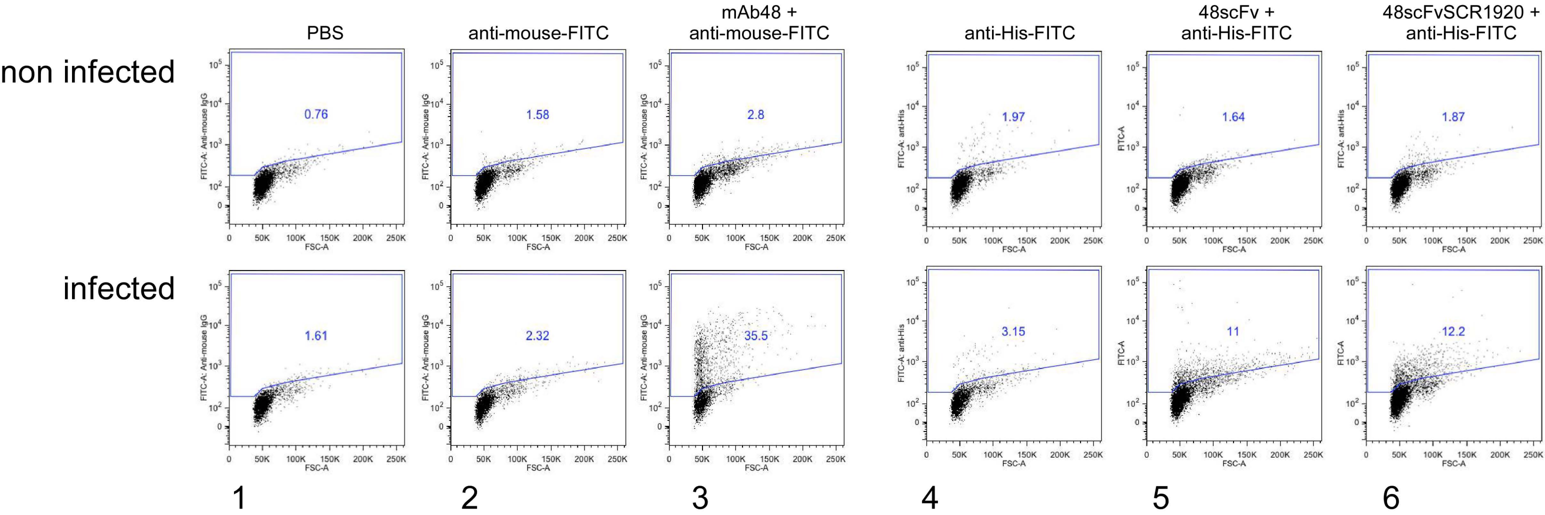

Column 1-3: Identification of infected *Mus dunni*-cell population using the monoclonal parent antibody clone 48 (mAb48). Column 4-6: The gate of mAb48-positive cells was used to identify functional single-chain Fv-constructs.
Non-infected *Mus dunni* cells served as negative control.

supplement data to figure 5:

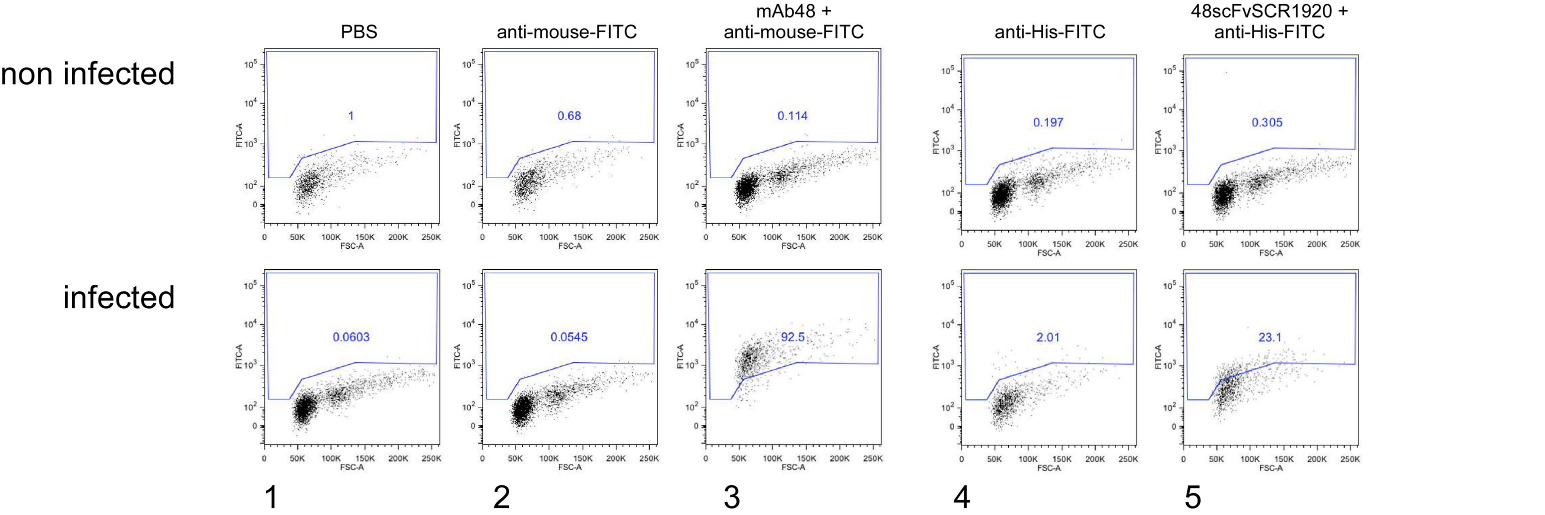

Column 1-3: Identification of infected *Mus dunni*-cell population using the monoclonal parent antibody clone 48 (mAb48). Column 4-5: The gate of mAb48-positive cells was used to identify functional single-chain Fv-construct.
Non-infected *Mus dunni* cells served as negative control.

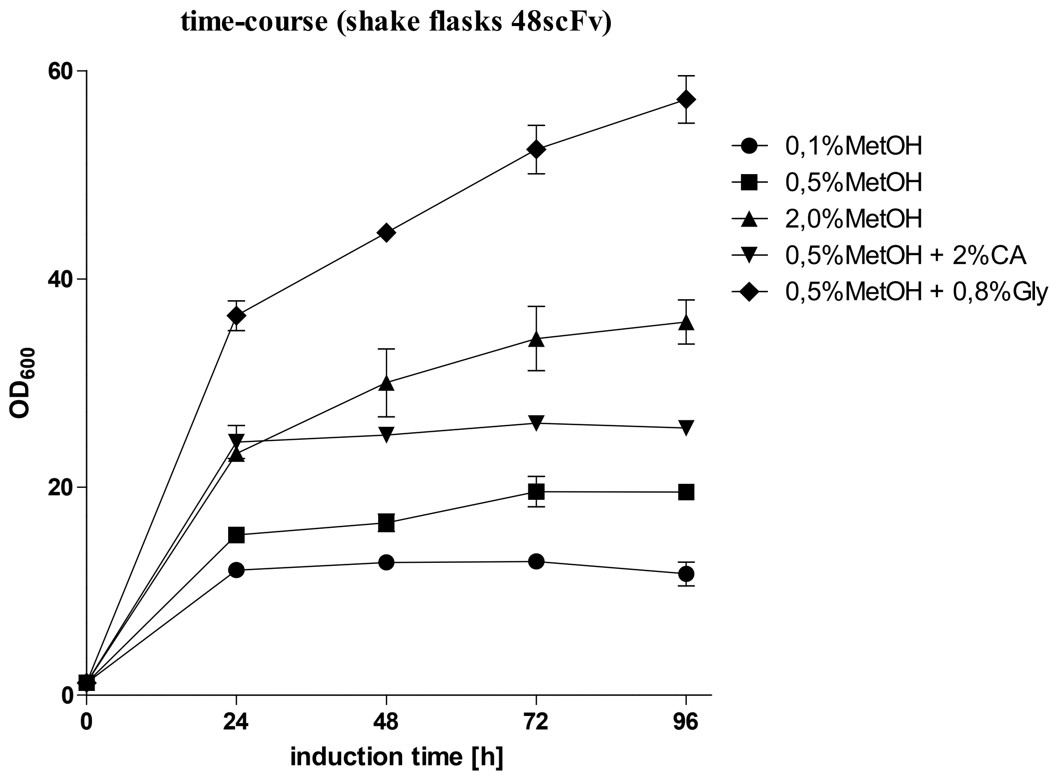

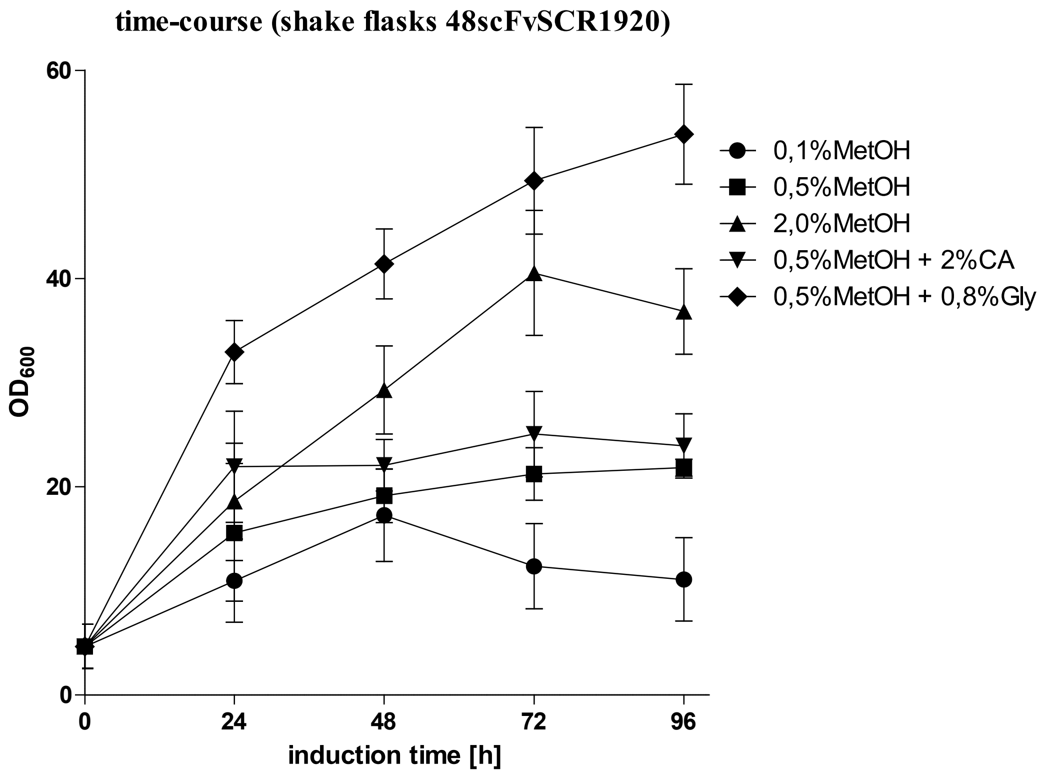

Biomass accumulation of *Pichia pastoris* expressing 48scFv and 48scFvSCR1920 in shake flasks under different conditions during 96h induction.

summarizing the cell viability of Pichia (shake flasks) after 96h induction

48scFv	dead cells (SD)	viability
0,1% MetOH	0,77(±0,15)	99,23
0,5% MetOH	0,53(±0,15)	99,47
2,0% MetOH	1,23(±0,15)	98,77
0,5% MetOH+2,0%CA	3,67(±0,15)	96,33
0,5% MetOH+0,8%Gly	0,27(±0,06)	99,73
48scFvSCR1920		
0,1% MetOH	1,13(±0,06)	98,87
0,5% MetOH	0,93(±0,06)	99,07
2,0% MetOH	9,53(±1,38)	90,47
0,5% MetOH+2,0%CA	1,67(±0,06)	98,33
0,5% MetOH+0,8%Gly	1,37(±0,15)	98,63
